# Ontogeny of Fetal Cardiometabolic Pathways: The Potential Role of Cortisol and Thyroid Hormones in Driving the Transition from Preterm to Near-Term Heart Development in Sheep

**DOI:** 10.3390/jcdd12020036

**Published:** 2025-01-21

**Authors:** Reza Amanollahi, Stacey L. Holman, Melanie R. Bertossa, Ashley S. Meakin, Kent L. Thornburg, I. Caroline McMillen, Michael D. Wiese, Mitchell C. Lock, Janna L. Morrison

**Affiliations:** 1 Early Origins of Adult Health Research Group, Health and Biomedical Innovation, UniSA: Clinical and Health Sciences, University of South Australia, Adelaide, SA 5001, Australia; reza.amanollahi@mymail.unisa.edu.au (R.A.); stacey.holman@unisa.edu.au (S.L.H.); melanie.bertossa@mymail.unisa.edu.au (M.R.B.); ashley.meakin@unisa.edu.au (A.S.M.); icmcmillen@outlook.com (I.C.M.); 2 Department of Medicine, Center for Developmental Health, Knight Cardiovascular Institute, Bob and Charlee Moore Institute of Nutrition and Wellness, Oregon Health & Science University, Portland, OR 97239, USA; thornbur@ohsu.edu; 3 Centre for Pharmaceutical Innovation, Clinical & Health Sciences University of South Australia, Adelaide, SA 5001, Australia; michael.wiese@unisa.edu.au

**Keywords:** heart development, cardiometabolic pathways, cortisol, thyroid hormones, preterm vs. near term, sheep

## Abstract

Understanding hormonal and molecular changes during the transition from preterm to near-term gestation is essential for investigating how pregnancy complications impact fetal heart development and contribute to long-term cardiovascular risks for offspring. This study examines these cardiac changes in fetal sheep, focusing on the changes between 116 days (preterm) and 140 days (near term) of gestation (dG, term = 150) using Western blotting, LC-MS/MS, and histological techniques. We observed a strong correlation between cortisol and T_3_ (Triiodothyronine) in heart tissue in near-term fetuses, highlighting the role of glucocorticoid signalling in fetal heart maturation. Protein expression patterns in the heart revealed a decrease in multiple glucocorticoid receptor isoforms (GRα-A, GR-P, GR-A, GRα-D2, and GRα-D3), alongside a decrease in IGF-1R (a marker of cardiac proliferative capacity) and p-FOXO1(Thr24) but an increase in PCNA (a marker of DNA replication), indicating a shift towards cardiomyocyte maturation from preterm to near term. The increased expression of proteins regulating mitochondrial biogenesis and OXPHOS complex 4 reflects the known transition from glycolysis to oxidative phosphorylation, essential for meeting the energy demands of the postnatal heart. We also found altered glucose transporter expression, with increased pIRS-1(ser789) and GLUT-4 but decreased GLUT-1 expression, suggesting improved insulin responsiveness as the heart approaches term. Notably, the reduced protein abundance of SIRT-1 and SERCA2, along with increased phosphorylation of cardiac Troponin I(Ser23/24), indicates adaptations for more energy-efficient contraction in the near-term heart. In conclusion, these findings show the complex interplay of hormonal, metabolic, and growth changes that regulate fetal heart development, providing new insights into heart development that are crucial for understanding pathological conditions at birth and throughout life.

## 1. Introduction

Preterm birth, defined as birth that occurs prior to 37 weeks of gestation in humans (term = 40 wks), has been associated with cardiovascular mortality in adulthood [1,2]. Preterm babies are more likely to be born too small, and studies have indicated an inverse association between birth weight and cardiovascular risk factors such as high blood pressure, dyslipidemia, glucose intolerance, and coronary heart disease [1,3,4]. The number of cardiomyocytes in the heart is set at birth because, during fetal development, the heart grows through the replication of cardiac cells; however, after birth, cardiomyocytes stop dividing [5,6,7]. Evidence suggests that preterm birth induces myocardial modeling, potentially altering its final structure and function [8]. Preterm birth also seems to cause a sudden decline in cardiomyocyte division, potentially reducing their final number [9]. In sheep, cardiomyocytes begin the process of becoming terminally differentiated at ~110 days of gestation (dG, term = 150), and at ~131 dG ~90% of cardiomyocytes are binucleated [6,7,10]. The regulation of cardiac growth and metabolism in the womb is critical to fetal wellbeing; however, despite advancements in neonatal care that improve the survival of preterm babies, the ontogeny of fetal cardiometabolic pathways is not fully understood. Thus the potential for new therapies to address metabolic compromise depends on a deeper understanding of fetal heart development. New information related to the normal metabolic maturation process from preterm to near term could help pinpoint key signalling pathways as targets for interventions in preterm babies affected by pregnancy complications.

Heart development is a complex process regulated by a variety of hormones and signalling molecules, among which cortisol and thyroid hormones play critical roles. Cortisol is the major glucocorticoid (GC) in humans and sheep and affects the conversion of the inactive thyroid hormone thyroxine (T_4_) to active triiodothyronine (T_3_) [11]. Fetal plasma cortisol concentration begins to rise during late gestation (~134 dG in sheep and ~30 wks in humans) to prepare the fetus for birth [12,13]. The peak of the prepartum surge in fetal plasma cortisol and circulating T_3_ occurs around the time of birth in sheep, which coincides with the cessation of proliferation and rise in binucleation in cardiomyocytes [14,15,16,17]. To initiate GC signalling, cortisol interacts with the glucocorticoid receptor (GR), regulating the expression of a variety of genes and proteins [18,19]. Multiple GR isoforms are derived from a single gene through alternative splicing and alternative translation initiation mechanisms [20]. In sheep, several GR isoforms have been identified in the placenta and lungs [21,22]; however, their expression and signalling pathways in the developing heart remain largely unknown.

Cardiometabolic changes across gestation are significant during this period when the heart shifts from using glucose and lactate for ATP production to fatty acids after birth. This shift is accompanied by a transition from glycolysis to oxidative phosphorylation (OXPHOS) to meet increasing cardiac energy demands [23,24]. In rodents, the transition to fatty acid metabolism occurs in the first week postnatally [25,26]; however, there is little information on large mammals. Recent swine studies suggest that this metabolic switch occurs before or around the time of birth [27,28]. Glucose transporters 1 and 4 (GLUT-1, and GLUT-4) are the most abundant isoforms in the heart, responsible for glucose uptake [29]. GLUT-1 is the predominant isoform in the embryonic and neonatal heart, while the insulin-dependent GLUT-4 is rapidly upregulated after birth, being the primary glucose transporter in the adult heart [29,30].

While rodent studies have shown that oxygen and nutrient availability play a crucial role in cardiometabolic maturation [31], they are not the sole contributing factors. Changes in the hormonal environment during late gestation, particularly involving cortisol and thyroid hormones, have also been shown to play a crucial role in the regulation of cardiac growth [5,14,32]. Five previous studies have explored the effects of intrafetal cortisol infusion on cardiac development in sheep; however, their findings were inconclusive, likely due to variations in gestational age, duration, and cortisol dosage [33,34,35,36,37]. Moreover, several interventional studies in sheep have emphasised the critical role of thyroid hormones in fetal heart maturation [11,14,38]. Hence, we hypothesised that significant molecular changes occur between preterm (116 dG) and near-term (140 dG, term = 150) in fetal sheep, particularly involving GR isoform expression and markers of cardiac growth, glucose metabolism, and contractility, driven by cortisol and thyroid hormones. Understanding these changes could provide insight into the mechanisms underlying fetal heart development during this critical transition period with an eye toward improving cardiovascular health in preterm babies affected by pregnancy complications.

## 2. Methods and Materials

### 2.1. Ethics and Animal Husbandry

All procedures were approved by the University of Adelaide Animal Ethics Committees (Ethics no. M/70/95, M/70/00) and comply with the Australian code of practice for the care and use of animals for scientific purposes. All investigators understood and followed the ethical principles outlined in Grundy et al. [39] and the principles of the 3Rs, specifically the reduction in the use of animals in research [40]. Fifteen pregnant Border Leicester×Merino and Merino ewes were housed in individual pens in animal rooms with a 12:12 h light/dark cycle. They received 100% of the metabolizable energy requirements once daily with water ad libitum.

### 2.2. Post-Mortem and Tissue Collection

At 116 or 140 days of gestation (dG, term = 150 d), the ewes were humanely killed with an intravenous overdose of sodium pentobarbitone (150 mg/kg, Virbac Pty Ltd., Peakhurst, NSW, Australia), and fetuses were delivered by hysterectomy, weighed, and body measurements were taken. The fetal hearts were removed, weighed, and a sample from the same region of the left ventricle (LV) from each animal was snap-frozen in liquid nitrogen, and stored at −80 °C, as well as fixed in 4% paraformaldehyde for subsequent molecular and histological analysis.

### 2.3. Quantification of Fetal Cardiac Protein Expression

Fetal LV tissue (~100 mg; preterm, *n* = 8, near term, *n* = 7) was cut and sonicated (John Morris Scientific, Adelaide, SA, Australia) in a lysis buffer containing Tris HCl (50 mM), NaCl (150 Mm), NP-40 (1%), Na Orthovanadate (1 mM), Na Fluoride (30 mM), Na Pyrophosphate (10 mM), EDTA (10 mM), and protease inhibitor (1 tablet/20 mL buffer; complete Mini, Roche). Samples were then centrifuged (12,400 rpm at 4 °C) for 14 min (Eppendorf Centrifuge 5415, Crown Scientific, Knoxfield, VIC, Australia). A Micro BCA Protein Assay Kit (PIERCE, Thermo Fisher Scientific Inc., Rockford, IL, USA) was used to determine the protein concentration of each sample. Bovine serum albumin (BSA; 2 mg/mL stock solution) was used for the standard curve. Extracted protein samples (5 mg/mL) were resolved via SDS-PAGE (12%) and stained with Coomassie blue to confirm a consistent concentration of protein for all diluted samples [41,42]. Equal concentration (5 mg/mL) and volume (15 µL) of each sample were subject to SDS-PAGE (10–15%) to detect specific proteins. Resolved proteins were then transferred onto a nitrocellulose membrane (Hybond ECL, GE Healthcare, Mascot, NSW, Australia), which were subsequently stained using Ponceau S solution (0.1% (*w*/*v*) in 5% acetic acid, Sigma–Aldrich, St. Louis, MA, USA), followed by imaging with ImageQuant LAS4000 (GE Healthcare, Melbourne, VIC, Australia). The membrane was washed with TBS (3 × 5 min) and then blocked with 5% BSA in Tris-Buffered Saline with 1% Tween (TBS-T) for 1 h at room temperature. The membranes underwent washes in TBS-T (3 × 5 min) and were cut according to the size of the proteins prior to incubation at 4 °C overnight with their respective primary antibodies. Target proteins included: Total OXPHOS (1:500, #ab110413, Abcam, Cambridge, UK), MitoBiogenesis cocktail (1:250, #ab123545, Abcam, Cambridge, UK), GLUT-4 (1:1000, #ab33780, Abcam, Cambridge, UK), GLUT-1 (1:1000, #sc-7903, Santa cruz biotechnology, Dallas, TX, USA), SERCA2 (1:1000, #ab137020, Abcam, Cambridge, UK), SIRT-1 (1:1000, #9475, Cell Signalling Technology, Danvers, MA, USA), Troponin I (1:1000, #4002, Cell Signalling Technology, Danvers, MA, USA), p-Troponin I(Ser23/24) (1:1000, #4004S, Cell Signalling Technology, Danvers, MA, USA), AS160 (1:1000, #2670S, Cell Signalling Technology, Danvers, MA, USA), p-AS160(Thr642) (1:1000, #4288S, Cell Signalling Technology, Danvers, MA, USA), Phospholamban (PLN; 1:1000, #14562S, Cell Signalling Technology, Danvers, MA, USA), p-Phospholamban (p-PLN; Ser16/Thr17) (1:1000, #8496S, Cell Signalling Technology, Danvers, MA, USA), Akt (1:1000, #9272S, Cell Signalling Technology, Danvers, MA, USA), p-Akt(Thr308) (1:1000, #9275S, Cell Signalling Technology, Danvers, MA, USA), IRS-1 (1:1000, #3194, Cell Signalling Technology, Danvers, MA, USA), p-IRS-1(Ser789) (1:1000, #2389S, Cell Signalling Technology, Danvers, MA, USA), GR (1:1000, #A303-491A, Bethyl Laboratories), mTOR (1:1000, #2972S, Cell Signalling Technology, Danvers, MA, USA), p-mTOR(Ser2448) (1:1000, #2971S, Cell Signalling Technology, Danvers, MA, USA), IGF-1R (1:1000, #3027S, Cell Signalling Technology, Danvers, MA, USA), PDK-4 (1:1000, #PA5-79800, Invitrogen, Waltham, MA, USA), PCNA (1:2000, #2586, Cell Signalling Technology, Danvers, MA, USA), p-P70 S6 Kinase(Thr389) (1:1000, #9205, Cell Signalling Technology, Danvers, MA, USA), P70 S6 Kinase (1:1000, #9202, Cell Signalling Technology, Danvers, MA, USA), p-FOXO1(Thr24) (1:1000, #9464, Cell Signalling Technology, Danvers, MA, USA), FOXO1 (1:1000, #9454, Cell Signalling Technology, Danvers, MA, USA), and NOX-2 (1:1000, #ab129068, Abcam, Cambridge, UK), as previously described [21,43,44,45]. All antibodies were diluted in 5% BSA in TBS-T. Membranes were again washed in TBS-T (3 × 5 min) before being incubated with the appropriate Horse Radish Peroxidase labelled secondary IgG antibody (1:2000, anti-mouse#7076, anti-rabbit#7054, Cell Signalling Technology) for 1 h at room temperature. Enhanced chemiluminescence using SuperSignal West Pico Chemiluminescent Substrate (Thermo Scientific, Waltham, MA, USA) was used to detect reactive proteins. The Western blot was imaged using ImageQuant LAS 4000 (GE Healthcare, Parramatta, NSW, Australia), and the protein abundance was quantified by densitometry using ImageQuant TL 8.1 software (GE Healthcare, Melbourne, VIC, Australia). The abundance of target proteins was then normalised to either Ponceau S or to a reference protein, Vinculin (1:2000, #18799S, Cell Signalling Technology, Danvers, MA, USA).

### 2.4. Quantification of Fetal Cardiac Concentration of Glucocorticoid and Thyroid Hormones

Tissue hormone concentrations were determined using liquid chromatography (LC; Shimadzu Nexera XR, Shimadzu, Kyoto, Japan) coupled to a SCIEX 6500+ Triple-Quad system (MS/MS; SCIEX, Framingham, MA, USA) using an adapted protocol [28,46,47]. Initially, a subset of LV tissue (preterm, n = 7, near term, n = 7) was homogenised in 500 μL 0.9% NaCl at 50 Hz for 2 min and then centrifuged at 12,000 *g* for 10 min at 4 °C. Supernatant (100 μL) was mixed with 300 μL acetonitrile containing 50 ng/mL internal standard (cortisol-9,11,12,12-d4; Toronto Research Chemicals, Toronto, ON, Canada), vortexed for 1 min and then centrifuged at 12,000× *g* for 10 min. The supernatant was transferred to a fresh Eppendorf tube and the remaining pellet was resuspended in 300 μL ethyl acetate, vortexed for 1 min, and then centrifuged at 12,000× *g* for 10 min. The supernatant was added to the acetonitrile, mixed via inversion, and then evaporated to dryness using the GeneVac EZ-2 Evaporating System (GeneVac, Ipswich, UK). Dried samples were reconstituted in 50% methanol and then injected into an ACQUITY UPLC BEH C18 Column (130Å, 1.7 µm, 2.1 mm × 100 mm (Waters Corp, Milford, MA, USA)). Mobile phases were 0.1% formic acid in water (A) and 0.1% formic acid in acetonitrile (B). The flow rate was 0.3 ml/min, and the mobile phase B was initially 10% and increased linearly to 90% over 10 min and then held at 90% for 2 min, after which it returned to 10% for 3 min prior to injection of the next sample. Hormone concentrations were calculated via integration with a standard curve that ranged from 0.05 to 100 ng/mL. Conditions for detection of analytes are as previously described [28,46,47,48].

### 2.5. Quantification of Fetal Cardiac Enzymatic Activity

A lactate dehydrogenase (LDH) assay kit (#ab102526, Abcam, Cambridge, UK) and citrate synthase (CS) assay kit (#CS0720, Sigma–Aldrich, St. Louis, MO, USA) were used to quantify the enzymatic activities of LDH and CS, respectively, in fetal left ventricle tissue (~100 mg; preterm = 8, near term = 7). The assays were performed according to the manufacturers’ protocols as previously described [28].

### 2.6. Quantification of Fetal Cardiac Glycogen and Collagen Staining

Paraformaldehyde fixed paraffin embedded blocks from a subset of fetal LV (preterm, n = 5, near term, n = 5) were sectioned at 5 μm (rotary microtome) onto SuperFrost slides (VWR International, United States). PAS (glycogen) and Masson’s trichrome (collagen) staining were performed by the University of Adelaide Histology Services. The slides were then scanned at 40× magnification using a NanoZoomer-XR (Hamamatsu, Japan) to generate whole-slide images. PAS slides were analysed using Fiji/Image J software (version 1.54f, NIH, Bethesda, MD, USA) using the colour saturation threshold tool at 20× magnification (five frames 1 mm apart). Masson’s trichrome slides were analysed using the VIS software suite version (Visiopharm 2020.08, Hoersholm, Denmark) using a custom threshold application at 10× magnification (whole slide). Correct quantification of the staining was confirmed by visual examination by a trained individual who was blinded to the treatment groups [49].

### 2.7. Quantification of Fetal Cardiac Ki67 Staining

Fetal LV tissue (preterm, n = 5, near term, n = 5; 5 μm thickness) was cut on a Leica HistoCore manual microtome (Leica Biosystems, Nussloch, Germany) from one embedded fixed tissue block per animal onto SuperFrost Plus slides (VWR International, Radnor, PA, USA). Slides were baked at 60 °C for 1 h followed by deparaffinisation and rehydration. After rehydration, endogenous peroxide activity was blocked with 3% hydrogen peroxide (Sigma–Aldrich; St. Louis, MO, USA), followed by heat-induced antigen retrieval (~20 min at 121 °C; 2100Retriver, Aptum Biologics, Southampton, UK) in citrate buffer (pH = 6.0). Slides were incubated overnight with the primary antibody (Ki67, 1:200, #M7240, Agilent Dako, Santa Clara, CA, USA) at 4 °C following incubation with non-immune serum (serum blocking solution; Thermo Fisher Scientific, Waltham, MA, USA). Negative control slides, prepared without the primary antibody, were utilised to verify the absence of nonspecific secondary antibody binding and reagent contamination. Additional controls included replacing the primary antibody with mouse serum (Sigma–Aldrich, St. Louis, MO, USA) and incubating it overnight at 4 °C. Positive cells were detected using the Metal Enhanced DAB Substrate Kit (#34065, Thermo Fisher Scientific, Waltham, MA, USA), and all sections were counterstained with Mayer’s haematoxylin (Sigma–Aldrich, St. Louis, MO, USA). The stained slides were scanned with a NanoZoomer-XR (Hamamatsu, Japan) and analysed using QuPath software (version 0.4.4, UK) to quantify the proportion of Ki67-positive cells in fetal LV tissue [49].

### 2.8. Statistical Analyses

All statistical analyses were performed using GraphPad Prism 10 (GraphPad Software, Inc., Boston, MA, USA). Some samples were not included in the analysis due to missing animal records (fetal parameters), systematic or technical errors (hormone assay), or missing fixed tissue samples (histology). Up to one outlier was removed from each group if identified using the Grubbs method (Alpha = 0.05). In the Western blot data, any non-quantifiable bands (due to a defect on the blots) were excluded from the analysis and are indicated on the blots with an X. Table and figure legends list the number of samples used for each analysis. Data were assessed for normality (Shapiro–Wilk test). Analysis of outcomes in preterm versus near-term animals was performed using an unpaired *t*-test if data were normally distributed; otherwise, a Mann–Whitney test was utilised. To assess the relationship between the two measures, simple linear regression was used. Three regressions were performed for each comparison, preterm, near-term term, and one including both groups (all data). The effect of sex was not evaluated due to the insufficient number of samples; however, sex is indicated in figures by different symbols. Data in figures and tables are presented as mean ± SD. A *p*-value of <0.05 was considered statistically significant.

## 3. Results

### 3.1. Fetal Heart and Body Growth

Body weight (*p* < 0.0001), crown-rump length (CRL; *p* < 0.0001), heart weight (*p* < 0.0001), and LV (*p* < 0.0011) and RV (*p* < 0.0001) weight were higher in the near-term compared to preterm fetuses (Table 1). However, total heart and LV and RV weight relative to the body weight were not different between the preterm and near-term fetuses (Table 1).

### 3.2. Hormone Concentrations of Fetal Cardiac Tissue

**Figure 1:**
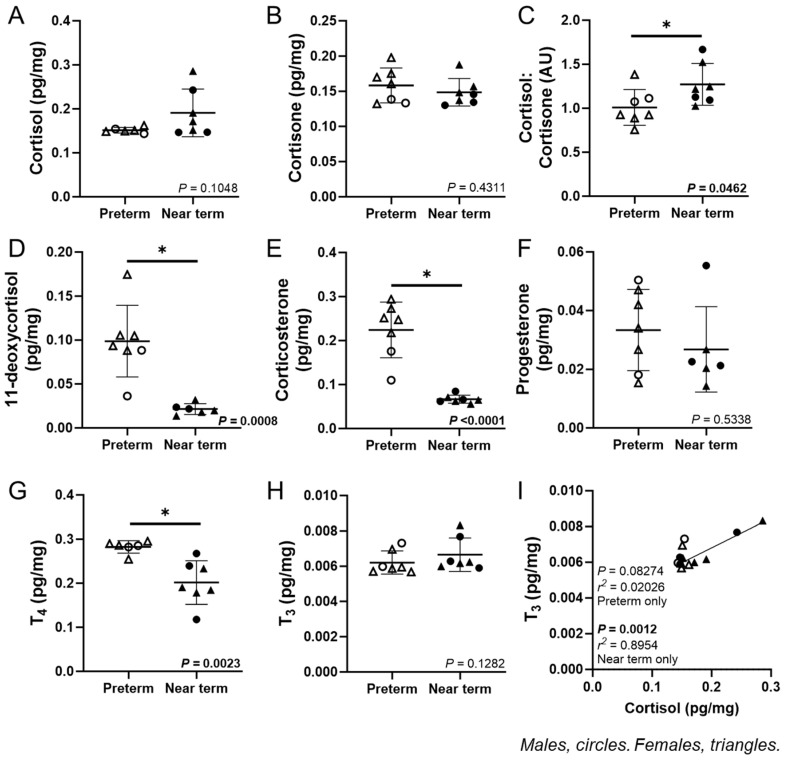
Hormone concentrations of fetal cardiac tissue. The fetal cardiac concentration of cortisol (**A**) and cortisone (**B**) were not different between preterm and near-term fetuses. The cortisol: cortisone ratio (**C**) was higher, while 11-deoxycortisol (**D**) and corticosterone (**E**) were lower with no change in progesterone (**F**) in the near-term compared to preterm fetuses. T_4_ (**G**) was lower with no change in T_3_ (**H**) concentrations in the near-term compared to preterm fetuses. In the near-term fetuses only, there were positive linear relationships of T_3_ with cortisol (**I**). Males (M) = circles, females (F) = triangles. preterm, left ventricle (LV) tissue from fetuses at 116 days of gestation (dG) (open symbols; hormone = 2M, 5F). Near-term, LV tissue from fetuses at 140 dG (closed symbols; hormone = 3M, 4F). One sample per animal was analysed via LC-MS/MS. Data were excluded due to a technical error in hormone concentration. Up to one outlier was excluded per group using the Grubbs method (Alpha = 0.05), when applicable. Data are expressed as mean ± SD and were analysed using either an unpaired *t*-test or simple linear regression. Data for progesterone, T_3_, and T_4_ failed the normality test and were consequently analysed using the Mann–Whitney test. (*) indicates a statistically significant difference between the groups. *p* < 0.05 was considered significant. AU: arbitrary unit.

The cardiac concentration of cortisol and cortisone was not different between preterm and near-term fetuses (Figure 1A,B). The cortisol: cortisone ratio (*p* = 0.0462) was higher, while 11-deoxycortisol (*p* = 0.0008), corticosterone (*p* < 0.0001), and T_4_ (*p* = 0.0023) were lower, with no difference in progesterone in the heart of near term compared to preterm fetuses (Figure 1C–F). The concentration of T_4_ (*p* = 0.0023) was lower, with no change in T_3_ in the near-term compared to preterm fetuses (Figure 1G,H). In the near-term fetuses only, there was a positive linear relationship between T_3_ and cortisol (*p* = 0.0012, *R*^2^ = 0.8954; Figure 1I).

### 3.3. Abundance of Glucocorticoid Receptor Isoforms in the Fetal Heart

**Figure 2:**
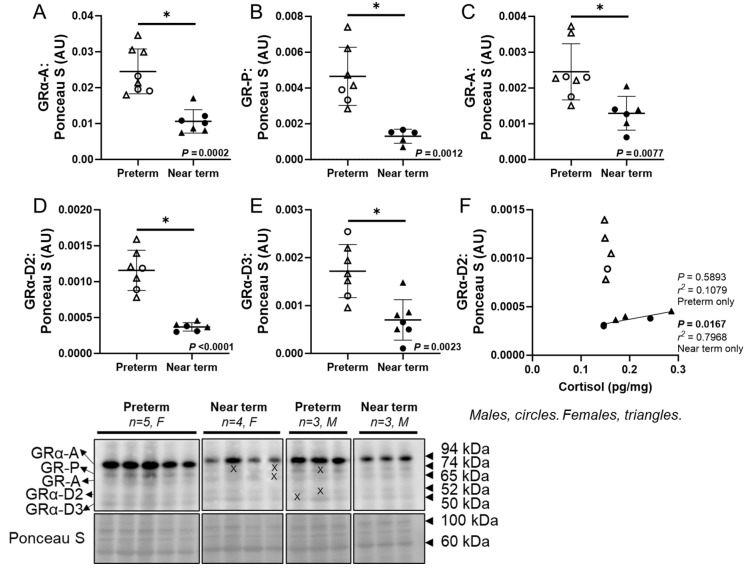
Abundance of glucocorticoid receptor isoforms in the fetal heart. The cardiac protein abundance of glucocorticoid receptors (GR) including GRα-A (**A**), GR-P (**B**), GR-A (**C**), GRα-D2 (**D**), and GRα-D3 (**E**) was lower in the near-term compared to preterm fetuses. In the near-term fetuses only, there was a positive linear relationship between cortisol and GRα-D2 (**F**). Males (M) = circles, females (F) = triangles. Preterm, left ventricle (LV) tissue from fetuses at 116 days of gestation (dG) (open symbols; protein = 3M, 5F; hormone = 2M, 5F). Near-term, LV tissue from fetuses at 140 dG (closed symbols; protein = 3M, 4F; hormone = 3M, 4F). One sample per animal was run per Western blot and LC-MS/MS. Data were excluded due to a technical error in hormone concentration. Up to one outlier was excluded per group using the Grubbs method (Alpha = 0.05) when applicable. Data were expressed as mean ± SD and were analysed using either an unpaired *t*-test or simple linear regression. (*) indicates a statistically significant difference between the groups. *p* < 0.05 was considered significant. AU: arbitrary unit. (X) indicates data excluded from analysis (due to a defect on the band/s).

The cardiac protein abundance of multiple GR isoforms including GRα-A (*p* = 0.0002), GR-P (*p* = 0.0012), GR-A (*p* = 0.0077), GRα-D2 (*p* < 0.0001), and GRα-D3 (*p* = 0.0023) were lower in the near-term compared to preterm fetuses (Figure 2A–E). In the near-term fetuses only, there was a positive linear relationship between cortisol concentration and GRα-D2 in the heart (*p* = 0.0167, R2 = 0.7968; Figure 2F).

### 3.4. Molecular Markers of Fetal Cardiac Growth

**Figure 3:**
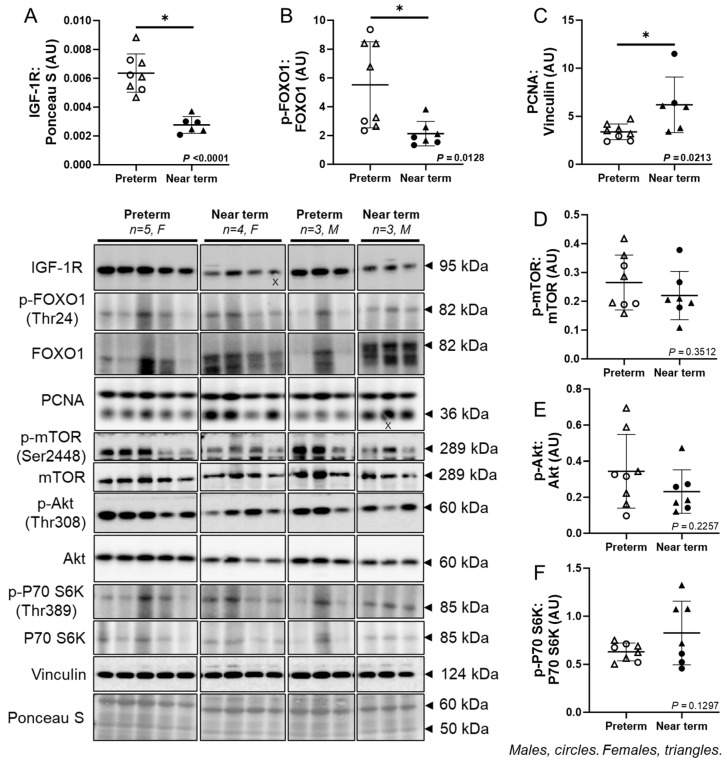
Molecular markers of fetal cardiac growth. The cardiac protein expression of IGF-1R (**A**) and p-FOXO1:FOXO1 ratio (**B**) was lower, while PCNA (**C**) was higher in the near term compared to preterm fetuses. There was no difference in the p-mTOR:mTOR ratio (**D**), p-Akt:Akt ratio (**E**), and p-P70 S6K:P70 S6K ratio (**F**) between the groups. Males (M) = circles, females (F) = triangles. Preterm, left ventricle (LV) tissue from fetuses at 116 days of gestation (dG) (open symbols; protein = 3M, 5F). Near-term, LV tissue from fetuses at 140 dG (closed symbols; protein = 3M, 4F). One sample per animal was run per Western blot. Up to one outlier was excluded per group using the Grubbs method (Alpha = 0.05), when applicable. Data are expressed as mean ± SD and were analysed either using an unpaired *t*-test or simple linear regression. (*) indicates a statistically significant difference between the groups. *p* < 0.05 was considered significant. AU: arbitrary unit. (X) indicates data excluded from analysis (due to a defect on the band/s).

The cardiac protein abundance of IGF-1R (*p* < 0.0001) and p-FOXO1:FOXO1 ratio (*p* = 0.0128) was lower, while PCNA (*p* = 0.0213) was higher in the near-term compared to preterm fetuses (Figure 3A–C). There was no difference in the p-mTOR:mTOR ratio, p-Akt:Akt ratio, and p-P70 S6K:P70 S6K ratio between the groups (Figure 3D–F).

### 3.5. Molecular Markers of Fetal Cardiac OXPHOS and Mitochondrial Content

**Figure 4:**
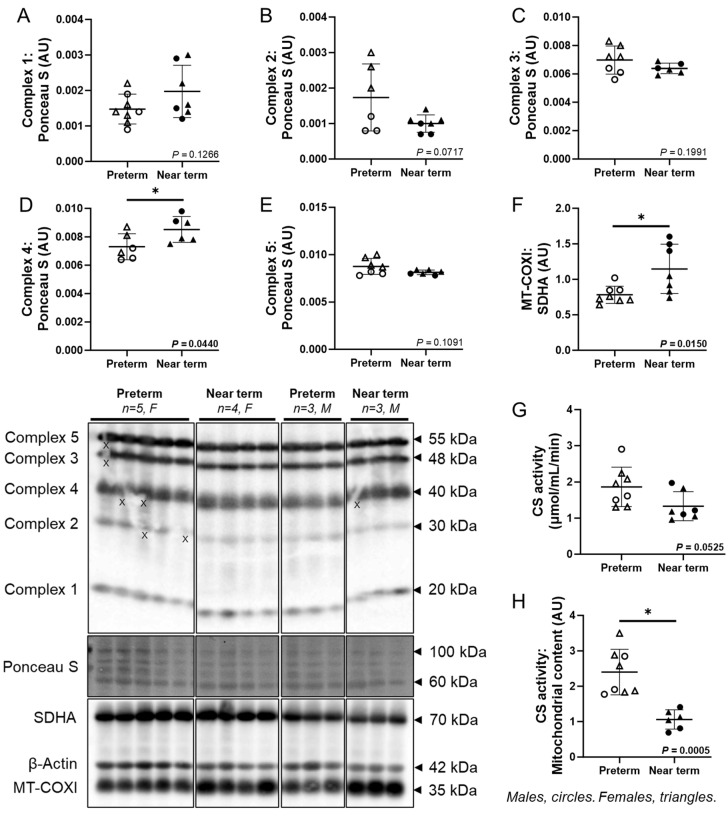
Molecular markers of fetal cardiac OXPHOS and mitochondrial content. The cardiac protein abundance of complex 4 (**D**) was higher in the near-term compared to preterm fetuses, while there was no difference in complex 1 (**A**), 2 (**B**), 3 (**C**), and 5 (**E**). The MT-COXI: SDHA ratio (**F**), a marker of mitochondrial content) was higher in the near-term compared to preterm fetuses. CS activity (**G**) did not differ between the groups, while CS activity: mitochondrial content ratio (**H**) was lower in the near-term compared to preterm fetuses. Males (M) = circles, females (F) = triangles. Preterm, left ventricle (LV) tissue from fetuses at 116 days of gestation (dG) (open symbols; protein/CS activity = 3M, 5F). Near-term, LV tissue from fetuses at 140 dG (closed symbols; protein/CS activity = 3M, 4F). One sample per animal was run per Western blot and CS activity. Up to one outlier was excluded per group using the Grubbs method (Alpha = 0.05), when applicable. Data are expressed as mean ± SD and were analysed using either an unpaired *t*-test or simple linear regression. (*) indicates a statistically significant difference between the groups. *p* < 0.05 was considered significant. AU: arbitrary unit. (X) indicates data excluded from analysis (due to a defect on the band/s).

The cardiac protein abundance of complex 4 (*p* = 0.0440) was higher in the near-term compared to preterm fetuses, while there was no difference in Complexes 1, 2, 3, and 5 (Figure 4A–E). MT-COXI: SDHA ratio (a marker of mitochondrial content) was higher (*p* = 0.0150), in the near-term compared to preterm fetuses (Figure 4F). CS activity did not differ between the groups, while CS activity: mitochondrial content ratio (*p* = 0.0005) was lower in the near-term compared to preterm fetuses (Figure 4G,H).

### 3.6. Molecular Markers of Fetal Cardiac Glucose Metabolism

**Figure 5:**
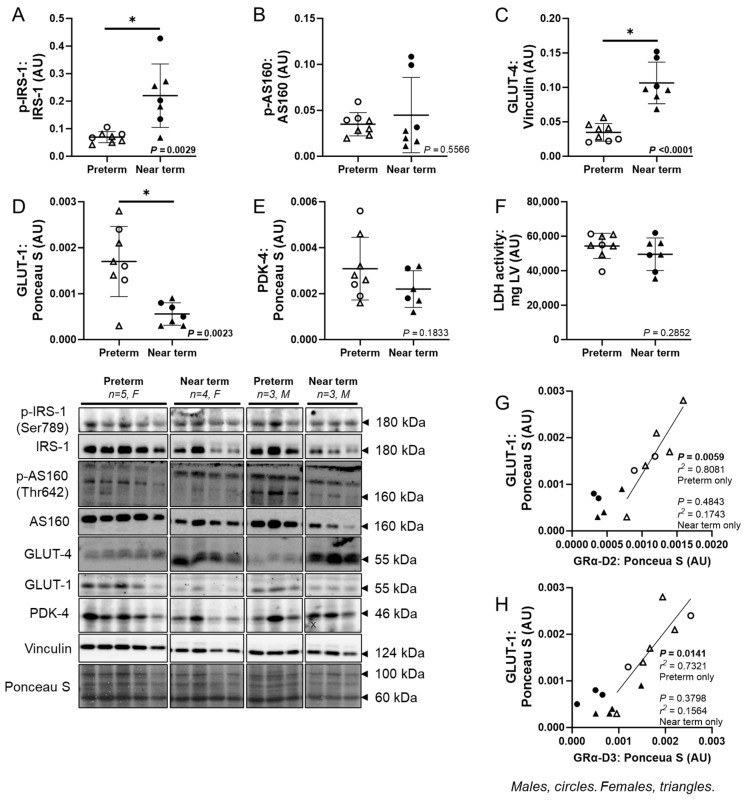
Molecular markers of fetal cardiac glucose metabolism. The ratio of p-IRS-1:IRS-1 ratio (**A**) and GLUT-4 (**C**) were higher, while the ratio of p-AS160:AS160 (**B**) was not different, and GLUT-1 (**D**) was lower in the near-term compared to preterm fetuses. The abundance of PDK-4 protein (**E**), and activity of LDH (**F**) were not different in preterm and near-term fetuses. In the preterm fetuses only, there were positive linear relationships between GRα-D2 and GLUT-1 (**G**), as well as GRα-D3 and GLUT-1 (**H**). Males (M) = circles, females (F) = triangles. Preterm, left ventricle (LV) tissue from fetuses at 116 days of gestation (dG) (open symbols; protein/LDH activity = 3M, 5F). Near-term, LV tissue from fetuses at 140 dG (closed symbols; protein/LDH activity = 3M, 4F). One sample per animal was run per Western blot and LDH activity. Up to one outlier was excluded per group using the Grubbs method (Alpha = 0.05), when applicable. Data are expressed as mean ± SD and were analysed using either an unpaired *t*-test or simple linear regression. Data for p-AS160:AS160 ratio failed the normality test and were consequently analysed using the Mann–Whitney test. (*) indicates a statistically significant difference between the groups. *p* < 0.05 was considered significant. AU: arbitrary unit. (X) indicates data excluded from analysis (due to a defect on the band/s).

The ratio of p-IRS-1:IRS-1 (*p* = 0.0029), as well as GLUT-4 (*p* < 0.0001), was higher, while p-AS160:AS160 was not different, and GLUT-1 (*p* = 0.0023) was lower in the near-term compared to preterm fetuses (Figure 5A–D). The abundance of PDK-4 protein and LDH activity were not different between the preterm and near-term groups (Figure 5E,F). In the preterm fetuses only, there were positive linear relationships between GRα-D2 and GLUT-1 (*p* = 0.0059, *R*^2^ = 0.8081), as well as GRα-D3 and GLUT-1 (*p* = 0.0141, *R*^2^ = 0.7321; Figure 5G,H).

### 3.7. Molecular Markers of Fetal Cardiac Contractility

**Figure 6:**
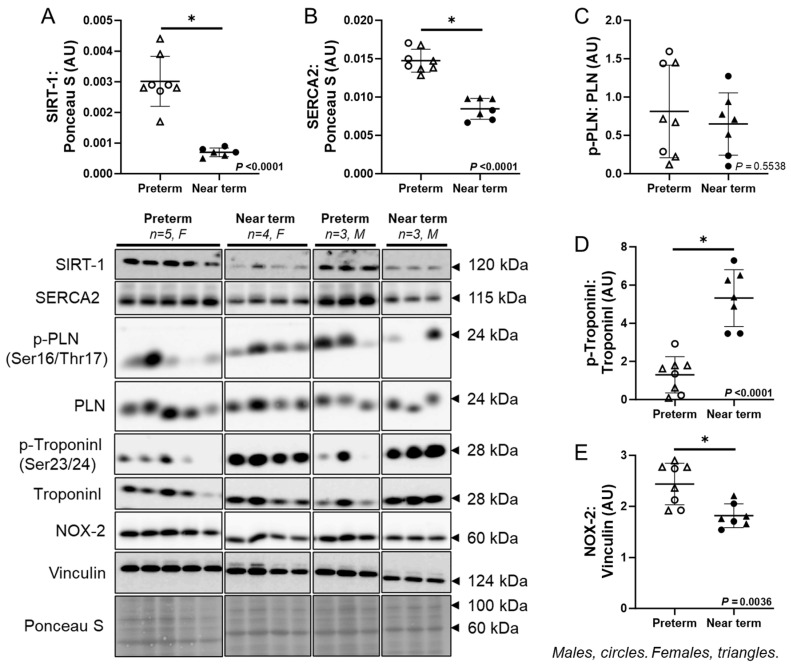
Molecular markers of fetal cardiac contractility. The expression of SIRT-1 (**A**) and SERCA2 (**B**) in cardiac tissue was lower, while there was no difference in the ratio of p-PLN:PLN (**C**) in the near-term compared to preterm fetuses. The ratio of p-TroponinI:TroponinI (**D**) was higher, while NOX-2 (**E**) was lower in near-term compared to preterm fetuses. Males (M) = circles, females (F) = triangles. Preterm, left ventricle (LV) tissue from fetuses at 116 days of gestation (dG) (open symbols; protein = 3M, 5F). Near-term, LV tissue from fetuses at 140 dG (closed symbols; protein = 3M, 4F). One sample per animal was run per Western blot. Up to one outlier was excluded per group using the Grubbs method (Alpha = 0.05), when applicable. Data are expressed as mean ± SD and were analysed using either an unpaired *t*-test or simple linear regression. (*) indicates a statistically significant difference between the groups. *p* < 0.05 was considered significant. AU: arbitrary unit.

The expression of SIRT-1 (*p* < 0.0001) and SERCA2 (*p* < 0.0001) in cardiac tissue was lower, while there was no difference in the ratio of p-PLN:PLN in the near-term compared to preterm fetuses (Figure 6A–C). The p-TroponinI:TroponinI ratio (*p* < 0.0001) was higher, while NOX-2 (*p* = 0.0036) was lower in the near-term compared to preterm fetuses (Figure 6D,E).

### 3.8. Fetal Cardiac Glycogen, Collagen, and Ki67 Staining

**Figure 7:**
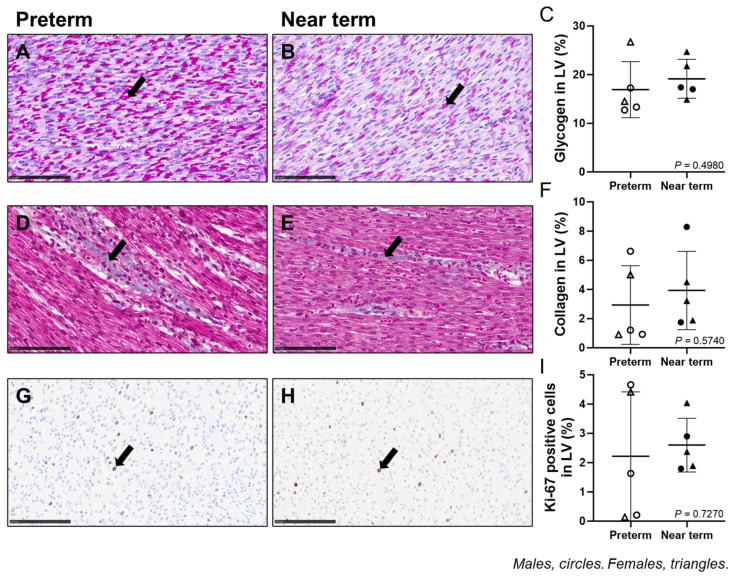
Fetal cardiac glycogen, collagen, and Ki67 staining: 20× magnification representative micrograph of glycogen staining using PAS (black arrow indicates glycogen stained in magenta) in preterm (**A**) and near term (**B**). 20× magnification representative micrograph of collagen staining using Masson’s trichrome (black arrow indicates collagen stained in blue) in preterm (**D**) and near term (**E**). 40× magnification representative micrograph of Ki67 staining using IHC (black arrow) in preterm (**G**) and near term (**H**). The fetal cardiac glycogen (**C**), collagen (**F**), and Ki67 (**I**) staining were not different between preterm and near-term fetuses. Males (M) = circles, females (F) = triangles. Preterm, left ventricle (LV) tissue from fetuses at 116 days of gestation (dG) (open symbols; histology/IHC = 3M, 2F). Near-term, LV tissue from fetuses at 140 dG (closed symbols; histology/IHC = 2M, 3F). One sample per animal was run per histology and IHC. A smaller subset of animals was included in this analysis due to missing fixed tissue samples. Scale bars = 100 μm. Data are expressed as mean ± SD and were analysed using an unpaired *t*-test. *p* < 0.05 was considered significant.

Cardiac glycogen, collagen, and Ki67 staining were not different between preterm and near-term fetuses (Figure 7).

## 4. Discussion

We examined cardiometabolic changes in preterm and near-term fetal sheep, revealing shifts in cardiac protein abundance, hormones, and metabolic pathways across gestation. A positive correlation between cortisol and T_3_ concentrations in the near-term heart suggests their combined role in regulating this transition as suggested by others [5]. Decreases in GR isoforms, IGF-1R, and FOXO1 phosphorylation, along with increased PCNA, are associated with cardiac maturation, while upregulation of OXPHOS complex 4, IRS-1 phosphorylation, and GLUT-4, alongside downregulation of GLUT-1 and NOX-2, points to a maturation of metabolic processes over the timeframe ranging from preterm to near term. Reduced SIRT-1 and SERCA2, along with increased phosphorylation of Troponin I, further reflect adaptations for more efficient cardiac function near term.

We found that the ratio of cortisol:cortisone in the heart increased across gestation from preterm to near term (116 vs. 140 dG). However, the concentrations of 11-deoxycortisol (a cortisol precursor) and corticosterone (a less dominant GC in large mammals) decreased with gestational age in the fetal heart. Most studies have focused on measuring fetal plasma concentrations of hormones, with limited data available on hormones in cardiac tissue, highlighting a gap in understanding the ontogeny of fetal heart development. Comparing fetal and maternal plasma concentrations of cortisol over 24 h between 127 and 142 dG in sheep, both mean plasma cortisol and its daily variation increased after 135 dG [50]. Cortisol prepares both mother and fetus for the process of birth, and there is a prepartum rise in plasma cortisol concentrations in mother and fetus in both sheep and humans [12,51,52,53,54,55]. In the current study, we found a strong positive correlation between cardiac concentration of cortisol and T_3_, but only in near-term fetuses. Cortisol induces deiodinase enzyme activity, which regulates the conversion of T_4_ to T_3_ [11]. In sheep, a surge in fetal plasma cortisol and T_3_ concentrations during late gestation (~135–145 dG, term = 150 d) coincides with the end of cardiomyocyte proliferation and an increase in binucleation [12,17]. In rats, a sharp rise in circulating corticosterone (analogous to cortisol in mice and rats) and T_3_ shortly after birth, around postnatal day 10 (P10), triggers a significant burst in cardiomyocyte proliferation by P15 [17,56].

This study revealed a decrease in the cardiac protein expression of multiple GR isoforms (GRα-A, GR-P, GR-A, GRα-D2, and GRα-D3) during fetal development, with a strong correlation observed between cortisol and GRα-D2 in only near-term fetuses. Multiple GR isoforms are generated from a single GR gene via alternative splicing and translation initiation [57]. While several GR isoforms have been identified in the human and sheep placenta and lungs [21,22,45], little is known about their expression in the developing fetal heart, their response to cortisol, and their role in cardiomyocyte maturation. To our knowledge, this is the first study comparing multiple cardiac GR isoforms between preterm and near-term fetal sheep. GRα-A is the primary functional isoform that regulates a variety of genes and proteins, via interaction with glucocorticoid response elements (GREs) [58]. GR-P and GR-A are involved in glucocorticoid resistance [59]. Unlike GRα-A, which remains in the cytoplasm without hormone and moves to the nucleus upon GC binding, the GRα-D isoform is present in the nucleus [20]. Moreover, nuclear GRα-D interacts with GRE-containing promoters of specific target genes independently of GC exposure [20,60]. The reduction in GR expression from preterm to near term may result from negative feedback regulation due to the rise in circulating cortisol concentration. Furthermore, the markers of cardiac proliferative growth including IGF-1R and p-FOXO1:FOXO1 ratio were also reduced from preterm to near term. Consistent with our findings, a cortisol infusion during late gestation (131–135 dG), mimicking the normal prepartum surge, suppressed cardiomyocyte proliferation in fetal sheep [34]. Insulin-like growth factor 1 (IGF-1) promotes cardiomyocyte proliferation [61], and elevated cortisol concentrations at term have been linked to reduced IGF-1 and IGF-2 expression in fetal sheep skeletal muscle [62], as well as decreased fetal cardiac expression of *IGF1R* mRNA [63]. Moreover, placental restriction increases *IGF2* and *IGF2R* mRNA expression in the sheep heart before and after birth, potentially activating IGF-1R and IGF-2R pathways, leading to delayed cardiomyocyte binucleation (IGF-1R-mediated event) and increased hypertrophy (IGF-2R-mediated event) in the fetal heart [64]. Forkhead box protein O1 (FOXO1) is expelled from the nucleus upon phosphorylation and is no longer active as a transcription factor. Consistent with our findings, phosphorylation of both FOXO1 and FOXO3 decreases postnatally in mouse hearts [65]. This is reflected by a reduction in phosphorylated FOXO1 abundance, which subsequently leads to decreased IGF1 protein expression during the first week after birth in mouse hearts in vivo [65]. Moreover, PCNA (a marker of DNA replication) protein abundance was increased in the near-term hearts, which may indicate an increase in cardiomyocyte binucleation, a process typically observed during maturation in late gestation [10]. Taken together, the observed decrease in markers of cardiac proliferative growth, along with the reduction in GR expression, may be linked to the process of cardiac maturation from preterm to near term. They may also be related to the observed correlation between cortisol and T_3_ in the near-term fetuses. To support this, the surge in cortisol and T_3_ begins before birth following birth in both humans and sheep, coinciding with the decline in cardiomyocyte proliferation during late gestation [14,15,16].

In the current study, the protein abundance of mitochondrial content and OXPHOS complex 4 were increased in the near-term fetal heart. OXPHOS consists of five complexes (1–5) located in the inner mitochondrial membrane, where electrons are passed through a series of redox reactions to generate ATP, the primary energy source for the heart [17]. Complex 4 (cytochrome c oxidase) is considered the final and rate-limiting step of the mitochondrial respiratory chain, serving as the regulatory centre of OXPHOS [66]. As the heart matures, there is a transition from glycolysis to OXPHOS, which is a more efficient pathway for ATP production [23]. This shift likely impacts complex 4 significantly during the transition from preterm to near-term heart development and may in part explain the selective increase in complex 4 while the other complexes remained unchanged towards near-term. Excess ATP may inhibit complex 4 production, thereby preventing the formation of reactive oxygen species (ROS), which are associated with numerous diseases [66,67]. However, this feedback inhibition of complex 4 can be disrupted by excessive workload and stress [66,68]. Notably, we observed a decrease in NOX-2 (a marker of ROS production) in the near-term heart, suggesting crosstalk between complex 4 and NOX-2 as the fetal heart matures. In the current study, although mitochondrial content increased in near-term heart, CS activity to mitochondrial content ratio was decreased. While total CS activity remained stable across four gestational time points in the mice placenta, the CS activity to mitochondrial content ratio decreased toward term [69], possibly due to senescence being associated with smaller and less metabolically active mitochondria [69]. Increasing mitochondrial content may compensate for this decline, ensuring proper function as the fetal heart prepares for the end of pregnancy.

Phosphorylated IRS-1 and GLUT-4 were more abundant, while GLUT-1 decreased as the fetal heart transitioned from preterm to near term. This aligns with previous studies indicating that GLUT-1 is the primary glucose transporter in fetal cardiomyocytes, with less GLUT-4 abundance [70,71]. After birth, GLUT-1 abundance decreases, while GLUT-4 increases, such that insulin-dependent GLUT-4 predominates in the adult heart [29]. Our findings indicate that as the fetal heart approaches the near term, it begins a metabolic transition by upregulating IRS-1 and GLUT-4, enhancing glucose uptake and increasing insulin responsiveness. This shift may be linked to the surge in plasma cortisol concentrations occurring at this stage. Additionally, we observed a strong positive correlation between GLUT-1 and both GRα-D2 and GRα-D3, but only in preterm hearts. This suggests that these GR isoforms may have the capacity to regulate glucose uptake during the preterm period when cortisol concentrations are low, as they can interact with GRE-containing promoters of specific target genes independently of GC exposure [20,60]. As expression of GRα-D2 and GRα-D3 decreases in near-term hearts, a concomitant reduction in GLUT-1 may promote a shift towards insulin-dependent, GLUT-4-mediated glucose uptake. However, further studies are needed to fully explore the role of specific GR isoforms during heart development.

With increasing gestational age, SIRT-1 and SERCA2 were decreased, while phosphorylated cardiac Troponin I increased. SIRT-1 protects the heart against apoptosis and plays a crucial role in supporting the survival of cardiomyocytes under stress [72,73]; however, its role in fetal heart development is not understood. SIRT-1 also has a significant role in cardiomyocyte proliferation by deacetylating p21 in mice [74]. This supports our findings that both SIRT-1 and IGF-1 abundance were elevated in the preterm fetal heart, where cardiomyocytes exhibit increased proliferation and enhanced protection against apoptosis compared to the near-term period. Cardiac contraction, essential for circulating blood throughout the body, is tightly regulated by the interaction between the sarcoendoplasmic reticulum calcium ATPase (SERCA) and phospholamban proteins [75]. SERCA has a higher concentration of the expressed protein in adults compared to fetuses, while mRNA levels remained consistent throughout human heart development [76]. However, consistent with our findings, a recent study reported a reduction in *SERCA* mRNA, no change in phospholamban phosphorylation, and an increase in phosphorylated cardiac Troponin I when comparing fetal and postnatal swine hearts [28]. Our findings suggest that as the fetus approaches term, increased phosphorylation of cardiac Troponin I may reduce calcium sensitivity, enabling more energy-efficient contractions in preparation for neonatal life. Increased cardiac troponin I and C protein expression across late gestation is consistent with reduced Ca^2+^ sensitivity and increased maximum Ca^2+^ activated force [77]. Thus, the decrease in SERCA2 may be a response to this adaptation.

### 4.1. Limitations

This study was designed to analyse key regulators of gestational changes that occur in the developing myocardium of the sheep fetus, which has many characteristics in common with human fetal development. We chose target genes/proteins that are known to be important in the metabolic maturation of the fetus but have never been studied at specific ages where they can be adequately compared. One limitation of this study is the sample size, which restricted our ability to investigate sex differences in the expression of proteins involved in cardiac growth and metabolism from preterm to near term. An additional limitation of the work is that we did not report functional changes that accompany maturation and we did not make a comprehensive study of all the regulatory proteins that may be important. However, we report herein changes in a number of proteins that highlight many important transitional processes that are not yet fully understood.

### 4.2. Future Directions

This study highlights several avenues for future research to further elucidate the mechanisms underlying fetal cardiac development. Investigating the precise role of individual GR isoforms in regulating glucose uptake, cardiomyocyte proliferation, and binucleation could provide deeper insights into the molecular pathways influenced by cortisol. Additionally, longitudinal studies examining the interplay between cortisol, T_3_, and IGF signalling during late gestation and early postnatal life are needed to understand their collective impact on cardiac maturation and function. The role of Troponin I and SERCA2 in fetal cardiac contractility and their implications for long-term cardiac health warrant further exploration. Moreover, understanding the crosstalk between OXPHOS complex 4 and NOX-2 in fetal cardiac mitochondria may help identify potential therapeutic targets for improving cardiac efficiency under stress conditions. Finally, comparative studies across species and developmental stages could uncover conserved and unique regulatory mechanisms, providing broader insights into fetal heart development.

## 5. Conclusions

In conclusion, this study highlights the critical cardiometabolic adaptations in the fetal sheep heart as it transitions from preterm to near term. The strong correlation observed between cortisol and T_3_ concentrations in the near-term heart underscores the critical role of GC signalling in driving essential physiological changes for a successful transition to postnatal life. The decline in cardiac expression of multiple GR isoforms, coupled with decreases in IGF-1R and p-FOXO1, and an increase in PCNA, indicates a shift in regulatory pathways that promote cardiomyocyte maturation. Furthermore, the increased protein abundance of mitochondrial content and OXPHOS complex 4, alongside alterations in GLUT-4 and GLUT-1, reflects a metabolic transition from reliance on glycolysis to a more efficient OXPHOS. Notably, the decrease in SIRT-1 and SERCA2 protein, combined with increased phosphorylation of cardiac Troponin I, suggests an adaptation towards energy-efficient contractions in the near-term heart. Overall, these findings shed light on the molecular changes in fetal heart development, particularly the role of cortisol and thyroid hormones in shifting cardiometabolic pathways from preterm to near term. This improves our understanding of how to study the impact of pregnancy complications on heart development and may help inform strategies to support cardiovascular health in preterm infants.

## Figures and Tables

**Table 1:** Fetal body and heart weights in preterm and near-term fetuses.

	Preterm (*n* = 8)	Near Term (*n* = 7)	*p* Value
Fetal Parameters			
Body weight (kg)	2.1 ± 0.3 (*n* = 7)	4.5 ± 0.4	<0.0001
Crown-rump length (CRL, cm)	45.2 ± 2.7 (*n* = 7)	56.9 ± 2.9	<0.0001
Heart weight (g)	16.8 ± 4.5	33.6 ± 6.4	<0.0001
Heart weight: body weight (g/kg)	8.0 ± 1.6 (*n* = 7)	7.3 ± 1.5	0.4334
LV weight (g)	9.2 ± 3.1 (*n* = 7)	16.7 ± 3.0 (*n* = 6)	0.0011
LV weight: body weight (g/kg)	4.2 ± 1.3 (*n* = 6)	3.7 ± 0.7 (*n* = 6)	0.4534
RV weight (g)	5.1 ± 0.9 (*n* = 7)	10.0 ± 1.7 (*n* = 6)	<0.0001
RV weight: body weight (g/kg)	2.3 ± 0.4 (*n* = 6)	2.2 ± 0.4 (*n* = 6)	0.6571

Data expressed as mean ± SD; were analysed using unpaired *t*-test. *p* < 0.05 was considered statistically significant. LV, left ventricle; RV, right ventricle. Animal numbers used within each subset of the study are shown in brackets. Data were excluded due to missing records.

## Data Availability

All data supporting the results are presented in the manuscript.

## Abbreviations

11βHSD	11β-hydroxysteroid dehydrogenases
Akt	Protein kinase B
AS160	Akt substrate of 160 kDa
CS	citrate synthase
CVD	Cardiovascular disease
dG	days of gestation
eNOS	Endothelial nitric oxide synthase
ETC	Electron transport chain
GLUT	Glucose transporter
GR	Glucocorticoid receptor
GC	glucocorticoid
IGF-1	Insulin-like growth factor-1
IGF-1R	Insulin-like growth factor-1 receptor
IHC	Immunohistochemistry
iNOS	Inducible nitric oxide synthase
IRS-1	Insulin receptor substrate-1
Ki67	Antigen Kiel 67
LDH	Lactate dehydrogenase
LV	Left ventricle
MR	Mineralocorticoid receptor
mTOR	Mammalian target of rapamycin
NOX-2	NADPH oxidase 2
OXPHOS	Oxidative phosphorylation
PAS	Periodic acid–Schiff
PCNA	Proliferating cell nuclear antigen
PDK4	Pyruvate dehydrogenase kinase-4
PGC-1α	Peroxisome proliferator-activated receptor-gamma coactivator-1alpha
PLN	Phospholamban
ROS	Reactive oxygen species
SERCA	Sarcoendoplasmic reticulum calcium ATPase
SIRT-1	Sirtuin-1
T_3_	Triiodothyronine
T_4_	Thyroxine
